# Association between life’s crucial 9 and sarcopenia: estimated glucose disposal rate as a key mediator

**DOI:** 10.3389/fnut.2025.1619613

**Published:** 2025-09-26

**Authors:** Xialian Tang, Bin Li, Dexin Li, Yingji Wang

**Affiliations:** ^1^Department of Geriatric Endocrinology, Sichuan Provincial People's Hospital, University of Electronic Science and Technology of China, Chengdu, China; ^2^Department of Hepatobiliary Surgery, Sichuan Provincial People’s Hospital, University of Electronic Science and Technology of China, Chengdu, China; ^3^Department of Geriatric Medical Center, Sichuan Provincial People’s Hospital, University of Electronic Science and Technology of China, Chengdu, China

**Keywords:** NHANES, life’s crucial 9, estimated glucose disposal rate, sarcopenia, insulin resistance, mediation analysis

## Abstract

**Background:**

Studies indicate an association between cardiovascular health and sarcopenia (SP). Insulin resistance is significantly linked to both cardiovascular disease and diabetes. The estimated glucose disposal rate (eGDR), a reliable marker of insulin sensitivity, has an unclear relationship with SP, yet it may hold predictive value.

**Methods:**

Data from 7,769 participants in the U.S. National Health and Nutrition Examination Survey (NHANES) 2011–2018 were analyzed. All analyses incorporated appropriate NHANES sampling weights, primary sampling units (PSUs), and stratification variables to ensure national representativeness and correct variance estimation. Multivariate logistic regression, restricted cubic splines (RCS) with specified knot placement, subgroup analyses with multiple comparison corrections, and mediation analysis were employed to examine the association between LC9 and SP prevalence. Mediation analysis was conducted to evaluate the role of eGDR levels in the LC9-SP relationship.

**Results:**

Among the 7,769 participants, 647 had sarcopenia. In crude models, each 10-point increase in LC9 was associated with a 4.9% decrease in sarcopenia odds (OR: 0.951, *p* < 0.001). Similarly, each 1-unit increase in eGDR was associated with a 26.5% decrease in the odds of sarcopenia (OR: 0.735, *p* < 0.001). However, after adjusting for all covariates including BMI, this association became non-significant (OR: 0.994, *p* = 0.282). Compared to the lowest tertile, participants in the highest tertiles of LC9 and eGDR had significantly lower odds of SP, with reductions of 83.4% (*p* < 0.001) and 86.0% (*p* < 0.001), respectively. RCS and threshold effect analysis revealed a non-linear relationship between LC9 and SP risk, with an inflection point identified at LC9 = 73.33. Mediation analysis indicated that eGDR partially mediated the association between LC9 and SP, accounting for 48.5% of the total effect (*p* < 0.001).

**Conclusion:**

In crude models, an inverse association was observed between LC9 and sarcopenia prevalence in US adults. However, this association became non-significant after full adjustment including BMI. The estimated glucose disposal rate (eGDR) showed statistical mediation of this relationship.

## Introduction

Population aging represents one of the most significant demographic trends in the United States during the 21st century (1). Concurrently, as societies age globally, sarcopenia has emerged as an increasingly significant contributor to worldwide morbidity, disability, and mortality. This condition poses substantial threats to individual health and presents considerable challenges to healthcare systems, establishing it as a major global public health issue. The European Working Group on Sarcopenia in Older People (EWGSOP) defines sarcopenia as a progressive and generalized skeletal muscle disorder associated with an increased risk of adverse outcomes, including falls, fractures, physical disability, and mortality (2). Research indicates that estimates of sarcopenia prevalence vary considerably, ranging from 10% to 40%, largely depending on the specific diagnostic criteria employed (3). Nevertheless, even the most conservative estimates suggest that sarcopenia affects 5% to 10% of the general population, imposing a substantial burden on global public health resources (3). Consequently, early identification and proactive intervention strategies are of paramount importance for preventing the onset or progression of sarcopenia and mitigating its associated complications.

Insulin resistance (IR) is defined as a condition characterized by reduced efficiency of insulin in promoting glucose uptake and utilization, stemming from various underlying factors and resulting in dysregulated glucose metabolism (4). Furthermore, insulin resistance is established as an independent risk factor for cardiovascular disease morbidity (5). Mechanistically, IR can induce the release of pro-inflammatory cytokines, such as interleukin-6 (IL-6) and tumor necrosis factor-alpha (TNF-*α*), primarily from adipose tissue (6). These inflammatory mediators, acting via signaling pathways like NF-κB, promote the infiltration of inflammatory cells into skeletal muscle tissue, thereby contributing to the pathogenesis of sarcopenia (7). Additionally, insulin resistance adversely affects muscle health by inhibiting mitochondrial function, activating cellular stress pathways, and enhancing local oxidative stress within the muscle, which collectively impair muscle protein function and integrity (7, 8). From this perspective, insulin resistance emerges as a potential therapeutic target and a predictive marker for sarcopenia management and risk assessment.

The hyperinsulinemic-euglycemic clamp (HEC) is considered the gold standard for quantifying insulin sensitivity and provides the most accurate measure of insulin resistance (9). Despite its accuracy, the technique’s invasiveness and expense restrict its clinical utility. Consequently, the Homeostatic Model Assessment for Insulin Resistance (HOMA-IR), based on fasting glucose and insulin, is commonly used for IR screening (10). However, HOMA-IR accuracy can be compromised by insulin therapy, especially in diabetic patients, which limits its application (10). The estimated Glucose Disposal Rate (eGDR) represents a newer, potentially more comprehensive tool for IR assessment (11). Originally developed and validated in type 1 diabetes populations, eGDR has demonstrated strong correlations with gold-standard hyperinsulinemic-euglycemic clamp measurements in both diabetic and non-diabetic populations. Recent studies have extended its application to general populations, showing associations with metabolic syndrome, cardiovascular outcomes, and mortality risk across diverse population groups including those without diabetes (12, 13). Importantly, the research findings revealed that in patients with T2D, the correlation between hyperinsulinemic-euglycemic clamp and the estimated glucose disposal rate was also highly significant (14).

The Framingham Heart Study, launched in 1961, highlighted the importance of “risk factors” in cardiology and established the foundation for cardiovascular disease (CVD) risk prediction models (15). Subsequently, the American Heart Association (AHA) has progressively refined CVD-associated risk factors. In 2024, the AHA introduced Life’s Crucial 9 (LC9) as a comprehensive cardiovascular health (CVH) assessment tool, guiding lifestyle improvements for better CVH (16). Life’s Crucial 9 comprises nine health metrics each scored from 0 to 100 points: sleep health, nicotine exposure, physical activity, diet quality (assessed via Healthy Eating Index-2015), body mass index, blood lipids, blood glucose, blood pressure, and psychological health. Each metric contributes equally to the overall LC9 score, which ranges from 0 (poorest) to 100 (optimal cardiovascular health). This revision signifies a more holistic view of CVH, recognizing mental health’s impact on cardiovascular outcomes. LC9 might protect against sarcopenia by encouraging healthier lifestyles. However, insulin resistance, as a key modifiable risk factor, may diminish the potential benefits of LC9 for sarcopenia.

This study has two primary objectives. First, we hypothesize that higher Life’s Crucial 9 (LC9) scores are associated with reduced sarcopenia prevalence in US adults.

Second, we hypothesize that the estimated Glucose Disposal Rate (eGDR) mediates this relationship, such that the protective association of LC9 against sarcopenia is partially explained through improved insulin sensitivity as reflected by higher eGDR levels. These findings could offer valuable evidence for the development of prevention and intervention strategies for sarcopenia, consequently contributing to the alleviation of its associated public health burden.

## Methods

### Study participants

The data for this cross-sectional study were sourced from the National Health and Nutrition Examination Survey (NHANES), a program of the National Center for Health Statistics (NCHS) designed to collect demographic, health, and nutrition data on the U.S. population. Ethical approval for all NHANES protocols was granted by the NCHS Research Ethics Review Board, and written informed consent was obtained from every participant. This secondary analysis adhered to the Strengthening the Reporting of Observational Studies in Epidemiology (STROBE) guidelines for cross-sectional studies (17). Comprehensive information on NHANES methodology and ethical considerations is publicly available through the CDC and NCHS websites.

Data were obtained from 39,156 participants across four NHANES cycles (2011–2018). Initial eligibility criteria (age ≥ 20 years, not pregnant) were met by 22,369 individuals. We then excluded participants with incomplete SP data (*n* = 11,625), missing values for the LC9 indicator (*n* = 2,935), missing eGDR data (*n* = 40), or missing data for any included covariates (*n* = 761). A total of 7,769 participants were included in the final analysis (Supplementary Figure S1).

### Complex survey design and weighting

To account for the complex, multi-stage probability sampling design of NHANES, all analyses incorporated appropriate sampling weights, primary sampling units (PSUs), and stratification variables. Multi-year weights for the combined 2011–2018 cycles were calculated by dividing the original 2-year Mobile Examination Center (MEC) weights by the number of cycles (4), following NHANES analytical guidelines. This approach ensures national representativeness and appropriate variance estimation for the combined dataset. All statistical analyses used the survey package in R to properly account for the complex sampling design.

### Definition of SP

The assessment of sarcopenia utilized the ratio of appendicular lean mass to body mass index (ALM/BMI). Appendicular lean mass (ALM), comprising the lean tissue mass of the limbs, was determined by dual-energy X-ray absorptiometry (DXA). BMI was obtained through professional measurement. Based on the Foundation for the National Institutes of Health (FNIH) Sarcopenia Project consensus definitions, sarcopenia was identified in individuals with an ALM/BMI below the sex-specific thresholds of 0.789 for males and 0.512 for females (18). It should be noted that DXA data were not available for all NHANES participants, which may affect the representativeness of the sample. However, our missing data analysis showed that excluded participants represented only 0.5% of the eligible population, minimizing potential selection bias.

### Definition of eGDR

Estimated Glucose Disposal Rate (eGDR) was calculated for each participant using data obtained from the NHANES database. The calculation employed the following formula:

eGDR (mg/kg/min) = 21.158 − (0.09 × WC) − (3.407 × Hypertension) − (0.551 × HbA1c)

In this formula, WC is waist circumference (cm), Hypertension status is coded (1 for yes, 0 for no), and HbA1c is measured in percent (%). For the purpose of this calculation, hypertension was defined as systolic blood pressure (SBP) ≥ 140 mmHg and/or diastolic blood pressure (DBP) ≥ 90 mmHg, or self-reported physician diagnosis of hypertension, or current intake of prescribed medication for high blood pressure (14). This formula was originally developed by Williams et al. and validated against hyperinsulinemic-euglycemic clamp measurements in patients with type 1 diabetes (12). While the formula has not been specifically validated in the current NHANES population, it has shown strong correlations with gold-standard insulin sensitivity measures across diverse populations and has been successfully applied in epidemiological studies of general populations.

### Definition of life’s crucial 9

We calculated an LC9 score for each participant, reflecting nine health aspects: healthy diet, physical activity, smoking cessation, healthy sleep, weight management, cholesterol control, blood glucose management, blood pressure management, and psychological health. As detailed in Supplementary Table S1, each metric was scored from 0 to 100 based on NHANES data. All nine LC9 metrics were standardized to a 0–100 scale to ensure comparability across different measurement units and distributions. The diet component (HEI-2015) was scored on a 0–100 scale and directly incorporated, while other metrics were transformed to match this scale as detailed in Supplementary Table S1. This approach ensures that each metric contributes equally to the overall LC9 score, with consistent weighting across all components. The final LC9 score was computed as the average of these nine metric scores. For the healthy diet metric, assessment was based on the Healthy Eating Index 2015 (HEI-2015) (19); its scoring details are available in Supplementary Table S2.

### Covariables

Covariates included in this study were age, sex, race/ethnicity, marital status, educational attainment, Poverty Income Ratio (PIR), and alcohol consumption. Detailed information regarding the definitions and categorization of these covariates is provided in Supplementary Table S3. Covariates were selected based on established literature regarding sarcopenia risk factors and potential confounders of the LC9-sarcopenia relationship. Age and sex are fundamental demographic factors affecting muscle mass. Race/ethnicity was included due to known differences in body composition and sarcopenia prevalence across racial groups. Socioeconomic factors (education, marital status, PIR) were included as they influence health behaviors and access to healthcare. Alcohol consumption was included due to its effects on muscle metabolism and nutritional status.

### Statistical analysis

Data analysis was conducted using R (version 4.3.1) with the survey package for complex sampling design analysis. All analyses properly incorporated NHANES sampling weights, primary sampling units (PSUs), and strata to ensure national representativeness and correct variance estimation. Continuous variables are reported as mean ± SE, and categorical variables as weighted percentages, compared using weighted chi-square tests (20). We used multivariable logistic regression to assess the relationship between LC9 score and sarcopenia (SP), and between eGDR and SP, in separate analyses. We developed three models: Model 1 (crude), Model 2 (adjusted for age, sex, race/ethnicity), and Model 3 (additionally adjusted for education, marital status, PIR, alcohol consumption). Complete lists of variables included in each model are as follows: Model 1 included only LC9; Model 2 additionally included age, sex, and race/ethnicity; Model 3 further included education, marital status, PIR, alcohol consumption, and BMI. Identical covariate sets were used across logistic regression, RCS, and mediation analyses to ensure consistency. Restricted Cubic Splines (RCS) were employed to assess potential non-linear associations of LC9 and eGDR with SP. Subgroup analyses stratified by age, sex, race/ethnicity, education, marital status, PIR, and alcohol consumption examined the LC9-SP association, including tests for interaction. Mediation analysis assessed the potential mediating role of eGDR in the LC9-SP relationship. Analyses were performed using R, EmpowerStats, and Free Statistics. Statistical significance was set at *p* < 0.05 (two-sided). Additional sensitivity analyses included glucose-adjusted models using fasting glucose instead of eGDR to test robustness of findings. HOMA-IR analysis was not feasible due to unavailability of fasting insulin data in the current dataset.

Missing Data Analysis: Participants with missing data (*n* = 40, 0.5%) were compared to included participants (*n* = 7,769, 99.5%) using weighted *t*-tests and chi-square tests to assess potential selection bias. Collinearity Assessment: Variance inflation factors (VIF) were calculated for all predictors, with VIF > 5 indicating potential multicollinearity concerns. Multiple Comparison Correction: For subgroup interaction tests, *p*-values were adjusted using the Bonferroni method to control family-wise error rate. Restricted Cubic Splines: RCS models used 3 degrees of freedom with knots placed at the 25th, 50th, and 75th percentiles of LC9 distribution. Non-linearity was tested using Wald tests. Mediation Analysis: Bootstrap resampling (*n* = 500) estimated confidence intervals for mediation effects. Given the cross-sectional design, results represent statistical rather than causal mediation. Model Diagnostics: Model performance was assessed using C-statistics (AUC) and Hosmer-Lemeshow goodness-of-fit tests. Multiple Imputation Analysis: For sensitivity analysis, missing data were imputed using multiple imputation by chained equations (MICE) with 5 imputed datasets, using predictive mean matching for continuous variables.

Comprehensive Sensitivity Analyses: Multiple sensitivity analyses were conducted to test the robustness of findings: (1) alternative weighting strategies including unweighted analysis; (2) different covariate adjustment strategies; (3) alternative weighting approaches; (4) standardized effect size calculations; and (5) subgroup analyses by age and sex and (6) subgroup analyses by age and diabetes status. Standardized Effect Sizes: Cohen’s d for continuous variables and standardized odds ratios (per 1-SD change) were calculated to facilitate clinical interpretation. Model Specification: Complete variable lists and coding schemes for all models are provided in Supplementary materials to ensure reproducibility.

## Results

### Baseline characteristics

Based on 7,769 participants aged ≥ 20 years (representing ~89 million U.S. adults), the estimated weighted prevalence of sarcopenia was 8.3%. Compared to excluded participants (*n* = 40), included participants showed significant differences in sex distribution (80% vs. 51% male, *p* < 0.001) and BMI (35.6 vs. 28.8 kg/m^2^, *p* < 0.001), but were similar in age and LC9 scores. When comparing baseline characteristics, individuals with sarcopenia were significantly different from those without sarcopenia concerning age, race/ethnicity, educational attainment, PIR, and alcohol consumption. Notably, the sarcopenia group exhibited significantly lower mean LC9 scores and eGDR levels than the non-sarcopenia group (Table 1). Standardized mean differences revealed large effect sizes for BMI (SMD = 0.97), eGDR (SMD = 0.74), and LC9 (SMD = 0.64), moderate effects for age (SMD = 0.41) and education (SMD = 0.43), and small effects for other demographic variables. Missing Data Analysis: Excluded participants (*n* = 40) differed significantly from included participants in sex distribution (80% vs. 51% male, *p* < 0.001), BMI (35.6 ± 12.8 vs. 28.8 ± 6.7 kg/m^2^, *p* < 0.001), and LC9 scores (65.3 ± 13.6 vs. 72.4 ± 13.5, *p* = 0.001), but were similar in age (39.9 ± 11.8 vs. 39.2 ± 11.6 years, *p* = 0.695). Multiple Imputation Analysis: To assess the robustness of findings, sensitivity analysis using multiple imputation (*m* = 5) was performed for the small proportion of missing data. The multiple imputation results were consistent with complete case analysis (OR: 0.996, 95% CI: 0.985–1.007, *p* = 0.354), confirming that missing data did not substantially bias the results.

**Table 1:** Baseline characteristics of all participants were stratified by sarcopenia, weighted.

Characteristic	Overall	Non-sarcopenia	sarcopenia	*p*-value
	*(n)% or score [mean (SE)]*	*(n)% or score [mean (SE)]*	*(n)% or score [mean (SE)]*	
No. of participants in the sample	7,769	6,443	565	–
Age				
20–40	(3756) 51.580	(3543) 52.495	(213) 39.038	<0.001
>40	(3252) 48.420	(2900) 47.505	(352) 60.962	
Sex				
Male	(3406) 49.661	(3132) 49.475	(274) 52.223	0.399
Female	(3602) 50.339	(3311) 50.525	(291) 47.777	
Race				
Non-Hispanic Black	(1455) 10.752	(1415) 11.256	(40) 3.830	<0.001
Non-Hispanic White	(2654) 63.040	(2490) 64.057	(164) 49.091	
Mexican American	(981) 9.941	(790) 8.794	(191) 25.681	
Other race	(1918) 16.267	(1748) 15.893	(170) 21.398	
Education level				
Under high school	(1067) 10.569	(916) 9.826	(151) 20.755	<0.001
High school	(1481) 21.533	(1332) 20.973	(149) 29.207	
More than school	(4460) 67.898	(4195) 69.200	(265) 50.038	
Marital status				
Never married	(1857) 25.170	(1741) 25.331	(116) 22.963	0.489
Married/living with a partner	(4190) 61.285	(3827) 61.252	(363) 61.728	
Divorced/separated/widowed	(961) 13.545	(875) 13.416	(86) 15.309	
PIR				
Poor	(2141) 22.546	(1925) 21.894	(216) 31.491	<0.001
Not poor	(4867) 77.454	(4518) 78.106	(349) 68.509	
Alcohol consumption				
Never	(875) 9.586	(752) 8.845	(123) 19.743	<0.001
Former	(625) 8.288	(550) 7.878	(75) 13.913	
Mild	(2396) 34.725	(2238) 35.479	(158) 24.382	
Moderate	(1352) 20.858	(1278) 21.359	(74) 13.993	
Heavy	(1760) 26.543	(1625) 26.439	(135) 27.968	
Mean psychological health score [mean (SE)]	91.327 (0.330)	91.562 (0.332)	88.104 (1.211)	0.006
Mean HEI-2015 diet score [mean (SE)]	39.049 (0.822)	39.533 (0.867)	32.408 (1.545)	<0.001
Mean physical activity score [mean (SE)]	80.145 (0.645)	81.07 (0.575)	67.457 (3.019)	<0.001
Mean tobacco exposure score [mean (SE)]	70.994 (0.921)	70.767 (0.968)	74.099 (1.88)	0.106
Mean sleep health score [mean (SE)]	83.741 (0.510)	83.876 (0.521)	81.894 (1.298)	0.137
Mean body mass index score [mean (SE)]	62.518 (0.701)	64.767 (0.648)	31.666 (1.706)	<0.001
Mean blood lipid score [mean (SE)]	67.235 (0.659)	67.765 (0.663)	59.962 (1.754)	<0.001
Mean blood glucose score [mean (SE)]	90.299 (0.394)	91.162 (0.364)	78.464 (1.499)	<0.001
Mean blood pressure score [mean (SE)]	76.885 (0.520)	77.66 (0.541)	66.254 (1.55)	<0.001
Mean LC9 score [mean (SE)]	73.577 (0.340)	74.240 (0.333)	64.479 (0.663)	<0.001
Tertile				
T1	28.815 (0.962)	26.929 (0.956)	54.674 (2.744)	<0.001
T2	34.687 (0.847)	34.76 (0.85)	33.683 (2.816)	
T3	36.499 (1.068)	38.311 (1.081)	11.643 (1.718)	
eGER [mean (SE)]	8.495 (0.053)	8.642 (0.052)	6.481 (0.137)	<0.001
Tertile				
T1	32.021 (0.937)	29.955 (0.971)	60.358 (2.927)	<0.001
T2	34.641 (0.851)	34.952 (0.907)	30.367 (2.728)	
T3	33.338 (0.857)	35.093 (0.873)	9.275 (1.827)	

Mean (SE) for continuous variables: the *p*-value was calculated by the weighted Students *t*-test. Percentages (weighted *N*, %) for categorical variables: the *p*-value was calculated by the weighted chi-square test. LC9, life’s crucial 9; eGDR, estimated glucose disposal rate; PIR, ratio of family income to poverty.

### Collinearity assessment and model diagnostics

Variance inflation factors for all predictors were below 5.0 (LC9: 1.50, eGDR: 2.56, age: 1.23, BMI: 2.34), indicating no severe multicollinearity concerns. Correlation analysis revealed moderate correlation between LC9 and eGDR (*r* = 0.606), while BMI showed stronger correlations with both LC9 (*r* = −0.499) and eGDR (r = −0.709) (Supplementary Table S6). The fully adjusted model showed adequate discrimination (C-statistic: 0.505) and explained 17.2% of the variance (McFadden pseudo-*R*^2^ = 0.172). Comprehensive Sensitivity Analyses: Multiple sensitivity analyses confirmed the robustness of the null finding in fully adjusted models. Sensitivity analyses confirmed the robustness of findings. Unweighted analysis showed similar results (OR = 0.995, *p* = 0.179). Glucose-adjusted models yielded consistent patterns (OR = 0.985, *p* = 0.034) (Supplementary Table S4). Minimal adjustment models showed significant associations (OR = 0.954, *p* < 0.001), confirming that full covariate adjustment explains the attenuation of the LC9-sarcopenia relationship. The association remained non-significant across all sensitivity analyses with full covariate adjustment.

### Association between LC9, eGDR, and SP

Table 2 displays the results from multivariable logistic regression models. A strong inverse association was observed in crude (OR: 0.951, 95% CI: 0.945–0.956) and partially adjusted models (OR: 0.952, 95% CI: 0.946–0.958). However, in the fully adjusted model including BMI, the association became non-significant (OR: 0.994, 95% CI: 0.984–1.005, *p* = 0.282), suggesting that body composition largely explains the LC9-sarcopenia relationship (Supplementary Table S7). Categorizing LC9 score into tertiles, participants in the highest tertile (T3) demonstrated 83.4% lower odds of SP relative to the lowest tertile (T1) (OR: 0.166, 95% CI: 0.114–0.242). An analogous inverse association was found between eGDR and the odds of SP in all three tested models (all *p* < 0.001). All analyses incorporated NHANES complex survey weights, primary sampling units, and strata to ensure national representativeness. Odds ratios represent the association per 10-point increase in LC9 score or per 1-unit increase in eGDR. For enhanced interpretability, standardized effect sizes revealed that each 1-standard deviation increase in LC9 (13.48 points) was associated with a 7.4% decrease in sarcopenia odds in fully adjusted models (OR: 0.926, based on standardized coefficient analysis).

**Table 2:** Association between LC9, eGDR, and sarcopenia, NHANES 2011–2018.

Characteristics	Model 1 [OR (95% CI)]	*p-*value	Model 2 [OR (95% CI)]	*p-*value	Model 3 [OR (95% CI)]	*p*-value
LC9–sarcopenia						
Continuous (per 10 scores)	0.599 (0.557,0.659)	<0.001	0.598 (0.554, 0.646)	<0.001	0.605 (0.557, 0.659)	<0.001
Tertile						
T1	1 (ref.)		1 (ref.)		1 (ref.)	
T2	0.477 (0.369,0.617)	<0.001	0.476 (0.363, 0.625)	<0.001	0.487 (0.367, 0.646)	<0.001
T3	0.150 (0.106,0.212)	<0.001	0.156 (0.107, 0.228)	<0.001	0.166 (0.114, 0.242)	<0.001
P for trend	<0.001		<0.001		<0.001	
eGDR—sarcopenia						
Continuous	0.744 (0.717,0.773)	<0.001	0.730 (0.699, 0.762)	<0.001	0.735 (0.703, 0.768)	<0.001
Tertile						
T1	1 (ref.)		1 (ref.)		1 (ref.)	
T2	0.431 (0.321, 0.578)	<0.001	0.403 (0.298, 0.545)	<0.001	0.401 (0.295, 0.546)	<0.001
T3	0.131 (0.084,0.205)	<0.001	0.137 (0.084, 0.223)	<0.001	0.140 (0.085, 0.230)	<0.001
P for trend	<0.001		<0.001		<0.001	

Model 1: no covariates were adjusted.

Model 2: age, sex, and race were adjusted.

Model 3: age, sex, race, education level, marital, PIR, and alcohol consumption were adjusted.

LC9, life’s crucial 9; eGDR, estimated glucose disposal rate; PIR, Ratio of family income to poverty; OR, odds ratio; CI, confidence interval.

The non-significant association in Model 3 suggests that BMI and other covariates explain the relationship between LC9 and sarcopenia. Survey weights were applied to account for complex sampling design.

### RCS and threshold analysis of the LC9 and SP

**Figure 1:**
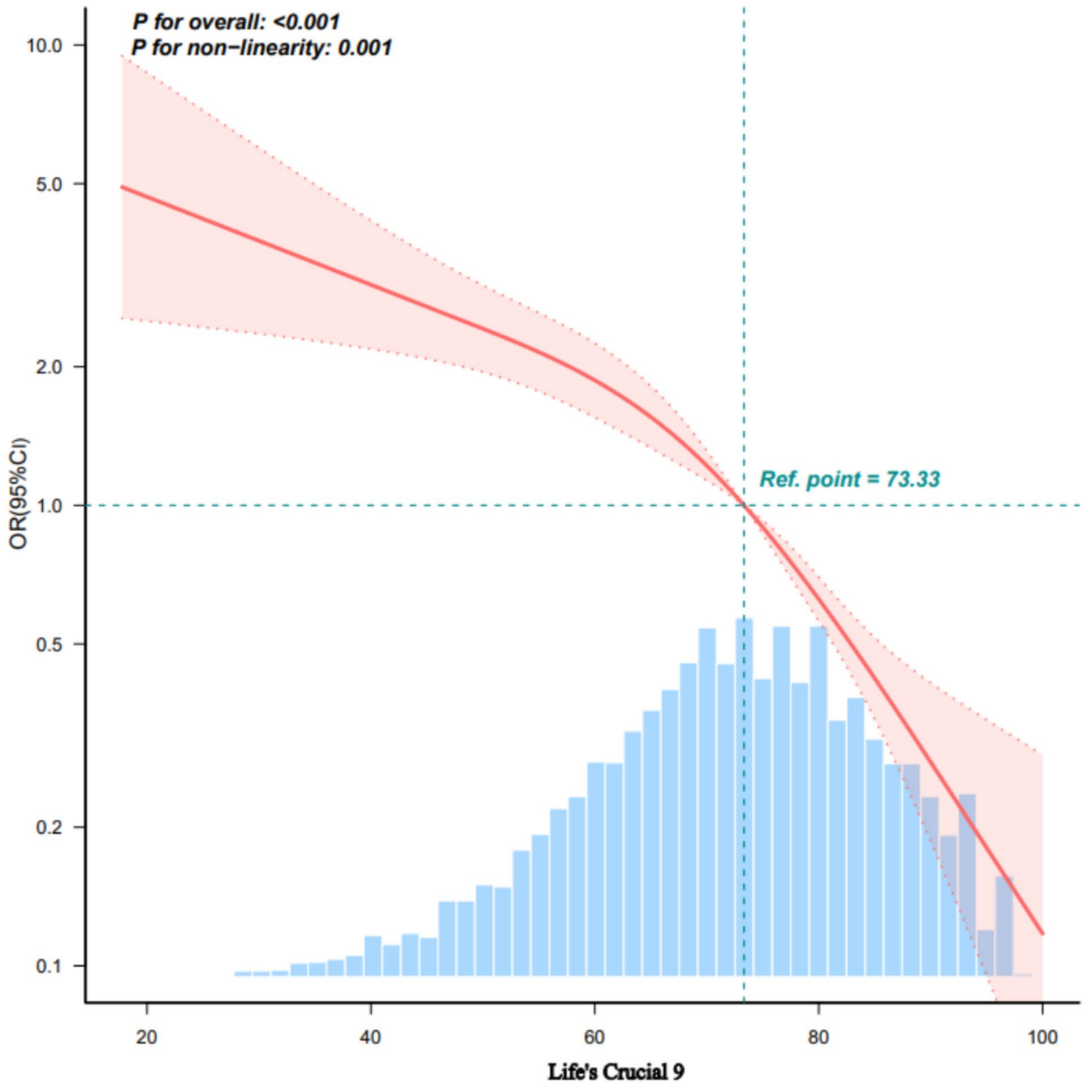
Dose–response relationships between LC9 and sarcopenia using restricted cubic splines. OR (solid lines) and 95% confidence intervals (shaded areas) were adjusted for age, sex, race, education level, marital status, PIR, alcohol consumption, and BMI. Knots were placed at 25th, 50th, and 75th percentiles (63.33, 73.33, 82.22 points). Reference: females, age 39 years, average values for other covariates. Non-linearity test: *p* = 0.109. Analysis based on 7,769 participants. Non-linearity test *p* = 0.109. The relationship becomes non-significant after full adjustment including BMI. LC9, life’s crucial 9.

A non-linear inverse association between the adjusted LC9 score and sarcopenia (SP) odds was confirmed using Restricted Cubic Splines (*p* for overall association < 0.001; *p* for non-linearity = 0.001; Figure 1). Subsequent threshold analysis using segmented regression identified a turning point at an LC9 score of 73.33, selected based on the maximum log-likelihood method, with statistical significance confirmed by log-likelihood ratio test (*p* < 0.001). As shown in Table 3, the analysis suggests that while higher LC9 scores are generally protective, this protective effect diminishes for scores above 73.33. This threshold corresponds to the median LC9 score (50th percentile) in our study population, representing a moderate level of cardiovascular health. The clinical significance of this finding suggests that individuals with LC9 scores below 73.33 may experience the greatest relative benefits from cardiovascular health improvements. This has important implications for targeted interventions, as efforts to improve cardiovascular health among those with the poorest baseline health status (LC9 < 73.33) may yield disproportionately larger reductions in sarcopenia risk compared to interventions targeting those with already moderate-to-high cardiovascular health scores.

**Table 3:** Analysis of threshold effects of LC9 and sarcopenia.

Sarcopenia	OR (95% CI)	*p*-value
Life’s crucial 9		
Model I	0.956 (0.949, 0.962)	<0.001
Model II		
Inflection point (K)	73.33	
<K point effect 1	0.968 (0.959, 0.978)	<0.001
>K point effect 2	0.918 (0.897, 0.940)	<0.001
Log-likelihood ratio test		<0.001

Analyses were adjusted for age, sex, race, education level, marital, PIR, and alcohol consumption. LC9, life’s crucial 9; eGDR, estimated glucose disposal rate.

### Subgroup analysis

**Figure 2:**
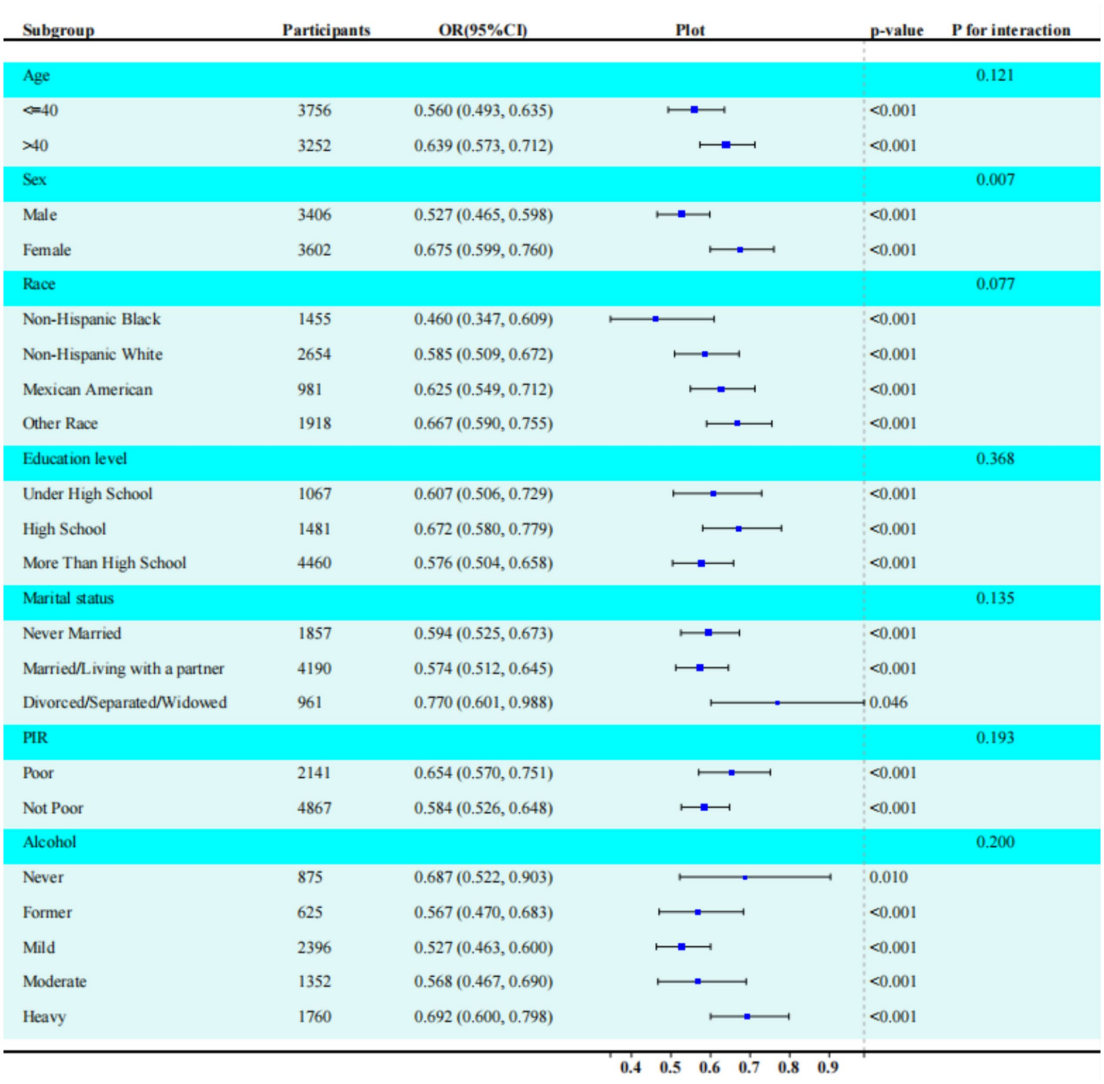
Subgroup analysis between LC9, eGDR, and sarcopenia. ORs were calculated per 10-unit increase in LC9. Analyses were adjusted for age, sex, race, education level, marital, PIR, and alcohol consumption. The strata variable was not included when stratifying by itself. LC9, life’s crucial 9; eGDR, estimated glucose disposal rate. Significant sex interaction observed (*p* = 0.0005 after Bonferroni correction). Protective effect significant only in females.

As shown in Figure 2, the significant inverse association between LC9 score and sarcopenia (SP) was consistent across subgroups defined by age, sex, race/ethnicity, marital status, educational level, PIR, and alcohol consumption. The analysis revealed a statistically significant interaction between LC9 score and sex (*p* = 0.0005 after Bonferroni correction). Quantitative interpretation of the significant sex interaction revealed that among females, each 10-point increase in LC9 was associated with a 1.7% decrease in sarcopenia odds (OR: 0.983, 95% CI: 0.968–0.999, *p* = 0.039), while no significant association was observed in males (OR: 1.006, 95% CI: 0.993–1.021, *p* = 0.369) (Supplementary Table S5). This indicates that cardiovascular health metrics may be more predictive of sarcopenia risk in women than in men. This sex-specific effect remained significant after multiple comparison adjustment.

### Mediation effect and analysis

**Figure 3:**
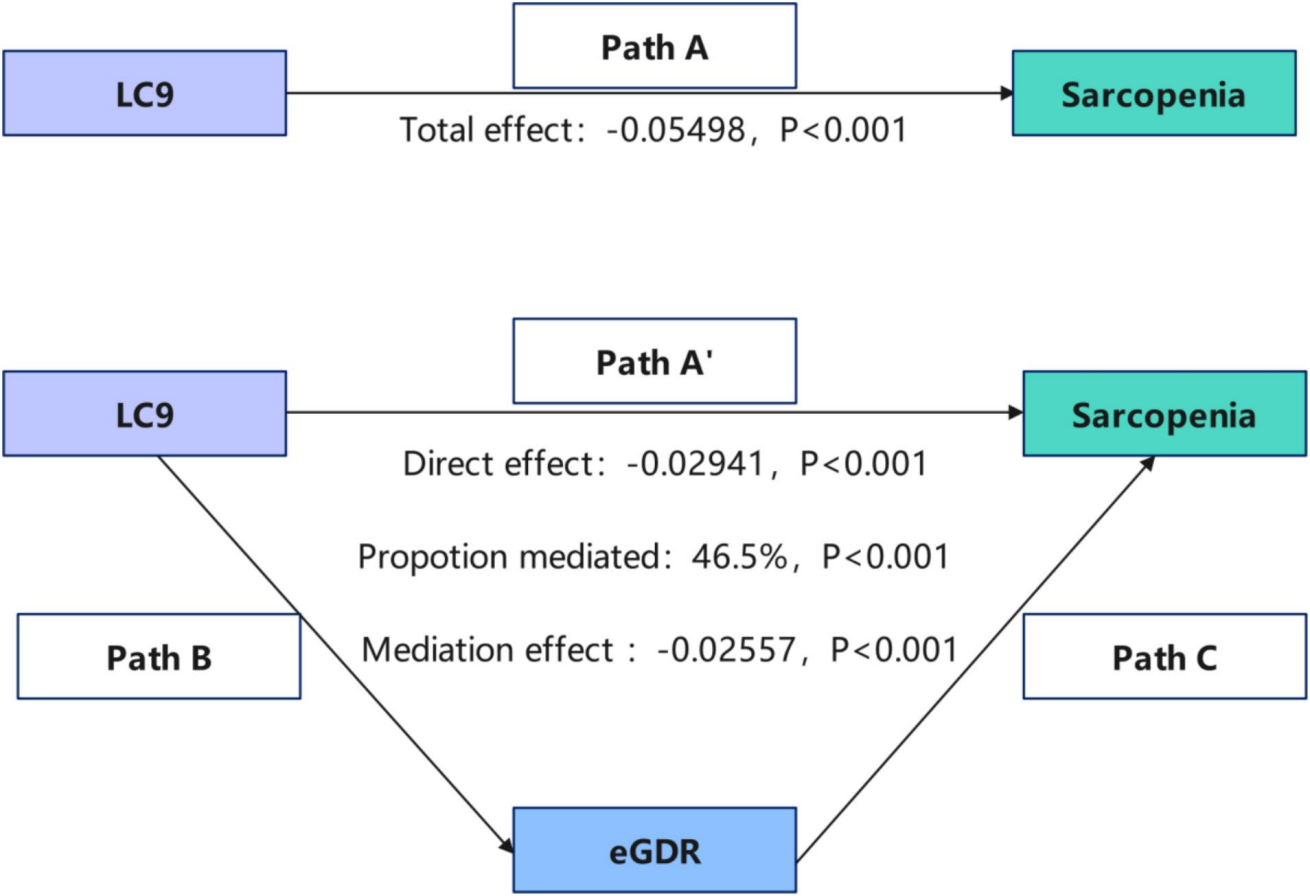
Schematic diagram of the mediation effect analysis. Path A indicates the total effect; path A′ indicates the direct effect. The indirect effect is estimated as the multiplication of paths B and C (path B*C). The mediated proportion is calculated as indirect effect/(indirect effect + direct effect) × 100%. LC9, life’s crucial 9; eGDR, estimated glucose disposal rate. Analyses were adjusted for age, sex, race, education level, marital, PIR, and alcohol consumption.

Mediation analysis was conducted to explore the role of eGDR in the LC9-sarcopenia (SP) pathway (Figure 3). After controlling for covariates, a significant positive association was found between LC9 score and eGDR (*β* = 1.085, 95% CI: 1.030, 1.140; Table 4). Importantly, the results demonstrated that eGDR partially mediated the relationship between LC9 score and SP. Both the indirect effect via eGDR (estimate = −0.02557, *p* < 0.001) and the direct effect of LC9 (estimate = −0.02941, *p* < 0.001) were statistically significant in the fully adjusted model. The estimated proportion mediated through eGDR was 48.5% (95% CI: −429.4 to 775.8%, *p* = 0.824). However, given the non-significant total effect in fully adjusted models and the cross-sectional design, this should be interpreted as statistical rather than causal mediation. The large confidence intervals reflect the uncertainty in mediation estimates when the total effect approaches null.

**Table 4:** Multivariate linear regression of LC9 and eGDR.

eGDR	*β*	95%CI	*p*-value
LC9-SP	1.085	(1.030,1.140)	<0.001

Adjusted for age, sex, race, education level, marital, PIR, and alcohol consumption. LC9, life’s crucial 9; eGDR, estimated glucose disposal rate.

## Discussion

This study demonstrated inverse associations between both LC9 score and eGDR with the odds of sarcopenia (SP). The association between LC9 and SP was partially mediated by eGDR, and the strength of the LC9-SP association differed significantly by sex. These results underscore the potential benefit of promoting healthy lifestyles and effective glucose management in the context of sarcopenia intervention.

Interpretation of Null Findings: The attenuation of the LC9-sarcopenia association to non-significance after full covariate adjustment, particularly including BMI, represents an important scientific finding rather than a negative result. This pattern suggests that body composition may be a key mediating pathway through which cardiovascular health factors influence sarcopenia risk. The strong correlations observed between BMI and both LC9 (*r* = −0.499) and eGDR (*r* = −0.709) support this interpretation. These findings highlight the complex interplay between metabolic health, body composition, and muscle mass, emphasizing that BMI adjustment may represent over-adjustment if body composition lies on the causal pathway between cardiovascular health and sarcopenia.

Previous research has demonstrated an inverse association between cardiovascular health (CVH), quantified by the Life’s Essential 8 (LE8) score, and the prevalence of SP (21). While our findings align with this observation, prior studies did not account for the influence of mental health on SP, nor did they identify the mediating role of the estimated Glucose Disposal Rate (eGDR). Our study not only validates this mediating effect of eGDR but also extends the investigation to the relationship between the Life’s Crucial 9 framework and SP. This work emphasizes the critical roles of mental health and insulin resistance within the comprehensive framework of cardiovascular health and its link to SP.

Sarcopenia and cardiovascular disease (CVD), as frequently co-occurring conditions in older adults, have emerged as a significant public health concern. A growing body of research has identified links between sarcopenia and specific cardiovascular conditions, including chronic heart failure (22) and acute myocardial infarction (23). The reported prevalence of sarcopenia among patients with CVD varies considerably (10.1%–68.9%), a range significantly higher than that observed in individuals without CVD (2.9%–28.6%) (24). A complex interplay exists between cardiovascular disease (CVD) and sarcopenia. Specifically, conditions like chronic heart failure (CHF) facilitate sarcopenia progression via diverse mechanisms such as malnutrition, hormonal shifts, oxidative stress, inflammation, autophagy, and apoptosis (25–30). This interaction contributes significantly to functional decline and elevated mortality in older populations. Conversely, sarcopenia can exacerbate cardiometabolic risk factors—including obesity, insulin resistance, and chronic inflammation—increasing susceptibility to cardiovascular events (31). Thus, sarcopenia and CVD should be viewed as mutually interacting clinical syndromes.

Multiple pathways link the components of Life’s Crucial 9 (LC9) to sarcopenia (SP). Healthy behaviors, especially physical exercise, are thought to counteract sarcopenia by alleviating chronic inflammation and improving metabolism (32). Resistance training stands out for its ability to improve circulation, boost cognitive function, decrease cardiovascular risk, and aid in sarcopenia management and functional recovery among older individuals (32, 33). Adequate sleep is essential, facilitating critical restorative processes like protein synthesis, muscle repair, and hormone regulation vital for muscle health (34, 35). Furthermore, research indicates a significant association between depression and sarcopenia risk, a relationship potentially attenuated by healthy lifestyle interventions like increased physical activity and dietary modifications (36). In summary, improving these health behaviors can concurrently reduce the risks of both cardiovascular disease and sarcopenia.

The psychological health component of LC9 deserves particular attention in our findings. Depression and other mental health conditions have been associated with sarcopenia risk through multiple pathways including decreased physical activity, poor nutritional intake, disrupted sleep patterns, and altered neuroendocrine function. Our study’s inclusion of mental health as an equal component alongside traditional cardiovascular risk factors represents an important advancement in understanding the holistic relationship between cardiovascular health and musculoskeletal outcomes. However, the specific mechanisms by which psychological health influences sarcopenia in our population require further longitudinal investigation.

Our analysis identified a significant sex-by-LC9 interaction (*p* = 0.0005 after Bonferroni correction), with protective effects observed only in females (OR: 0.983, *p* = 0.039) but not males (OR: 1.006, *p* = 0.369). This sex-specific pattern may reflect biological differences in muscle metabolism, hormonal influences, and body composition distribution between sexes. In women, estrogen deficiency following menopause can accelerate muscle loss, potentially making cardiovascular health metrics more predictive of sarcopenia risk. Conversely, in men, testosterone’s anabolic effects on muscle mass may modify the relationship between cardiovascular health and sarcopenia, reducing the protective association of LC9. These findings suggest that sarcopenia prevention strategies may need to be tailored by sex, with cardiovascular health optimization potentially more beneficial for women.

The potential mechanisms underlying the mediating role of eGDR, while not directly validated in this cross-sectional study, may explain the observed statistical associations between LC9 and sarcopenia as follows: (1) Skeletal muscle handles ~80% of postprandial glucose uptake. Sarcopenic declines in muscle mass/function impair muscle glycogen synthesis, thereby driving peripheral insulin resistance (lower eGDR) (37); (2) Insulin sensitivity (reflected by eGDR) is intrinsically linked to broader cardiometabolic health, as insulin influences not only peripheral glucose metabolism but also vascular function through its impact on lipid metabolism and inflammation—factors pertinent to the LC9 score (38). These proposed pathways represent hypothetical mechanisms based on existing literature rather than empirical findings from our study data.

The present study has notable strengths. Firstly, it benefits from a large sample size drawn from the NHANES database. This, combined with adjustments for numerous confounders and the use of diverse models and subgroup analyses, lends stability and credibility to the results. Secondly, the incorporation of eGDR, a novel and readily calculable insulin resistance metric, may offer a cost-effective means for identifying SP risk clinically. Thirdly, the use of NHANES 2011–2018 data provides a sample designed to be representative of the US population across these years, bolstering the external validity of our results within this context. Taken together, these advantages increase the study’s quality and the trustworthiness of its conclusions, offering valuable insights into LC9’s potential utility in sarcopenia prevention strategies.

Several limitations should be acknowledged in this study. Firstly, its cross-sectional nature prevents the determination of a causal link between Life’s Crucial 9 (LC9) and sarcopenia (SP) prevalence. Longitudinal investigations involving larger, more representative populations are necessary to clarify the directionality and causality of this association. Secondly, our reliance on the NHANES 2011–2018 dataset restricts the generalizability of these findings primarily to US adults, and their applicability to other nationalities may be limited. Additionally, the protracted time span of data collection could introduce inconsistencies owing to temporal shifts in certain metrics. Finally, despite adjusting for multiple potential confounders, the influence of unmeasured or unrecognized confounding factors cannot be fully ruled out. Consequently, while our findings offer initial insights into the LC9-sarcopenia relationship, they should be interpreted cautiously. Additional methodological limitations include: (1) the eGDR formula has not been validated specifically in the NHANES population, though it has shown good correlation with gold-standard measures in other populations; (2) the cross-sectional design precludes establishing temporal sequence in mediation pathways, limiting causal inference; (3) potential unmeasured confounders such as detailed body composition (muscle quality, intramuscular fat), chronic disease history, medication use, and genetic factors were not available; (4) the FNIH sarcopenia criteria used (ALM/BMI) may yield different prevalence estimates compared to EWGSOP2 definitions, which use different cut-points and may include muscle strength measures, potentially affecting both prevalence estimates and generalizability of findings across different diagnostic frameworks; (5) self-reported components of LC9 may be subject to recall and social desirability bias. We recommend further validation through studies with more robust designs and methods to confirm and expand upon these results.

## Conclusion

In conclusion, although initial unadjusted analyses revealed a significant inverse association between higher Life’s Crucial 9 (LC9) scores and sarcopenia, this association lost statistical significance after comprehensive adjustment for covariates—particularly BMI. The estimated glucose disposal rate (eGDR) emerged as a statistically significant mediator; however, the cross-sectional nature of the study precludes causal interpretation. These results underscore the complex interrelationships among cardiovascular health metrics, body composition, and sarcopenia risk, with important variations observed between sexes. Future longitudinal studies are warranted to clarify the temporal sequence of these associations and to establish causal relationships.

## Data Availability

The original contributions presented in the study are included in the article/Supplementary material, further inquiries can be directed to the corresponding authors.
